# The complete mitochondrial genome and phylogenetic analysis of Yanglong yak (*Bos grunniens*)

**DOI:** 10.1080/23802359.2021.1910086

**Published:** 2021-04-15

**Authors:** Shaoke Guo, Xiaoyun Wu, Rende Song, Xita Za, Qingzhang Zhao, Jiye Li, Haiqing Ma, Fude Wu, Chunnian Liang, Jie Pei, Xian Guo

**Affiliations:** a Key Laboratory of Yak Breeding Engineering of Gansu Province, Lanzhou Institute of Husbandry and Pharmaceutical Sciences, Chinese Academy of Agricultural Sciences, Lanzhou, People’s Republic of China; b Animal Disease Prevention and Control, Center of Yushu Tibetan Autonomous Prefecture in Qinghai Province, Yushu, People’s Republic of China; c Animal Husbandry and Veterinary Station of Qilian County in Qinghai Province, Qilian, People’s Republic of China; d Animal Husbandry and Veterinary Station of Xitan Township in Menyuan County in Qinghai Province, Menyuan, People’s Republic of China; e Datong Cattle Farm in Qinghai Province, Xining, People’s Republic of China

**Keywords:** Illumina sequencing, interspecific hybridization, mitogenomics, iterative mapping

## Abstract

In this study, we assembled the complete mitochondrial genome of Yanglong yak (*Bos grunniens*) from Illumina sequencing reads. The mitochondrial genome is 16,323 bp long with an A + T-biased nucleotide composition, and encodes 13 protein-coding, 22 tRNA, and two rRNA genes along with a noncoding control region. In addition, its gene order is identical to those of the previously published mitochondrial genomes of its congeners. Phylogenetic analysis indicates that this breed is closely related to Datong yak, Pamir yak, Tianjun yak, polled yak, Seron yak, Sunnan yak, a series of Domestic Yak and wild yak, followed by Jinchuan yak and Gannan yak, and slightly far away from *Bison* and *Bos taurus*.

The genetic characteristics of the mitochondrial genome are one of the important means to study molecular biology (Lorenzo et al. 2016). The animal mitochondrial genome is characterized by simple structure and easy detection. Moreover, as the mitochondrial genome is inherited from the mother line, it is very beneficial to study the genetic evolution and phylogeny of the population. For centuries, yaks have been maintained by local nomadic pastoralists for transportation, milk, butter, meat, and other byproducts (Leslie and Schaller 2009; Qiu et al. 2012). *Bos grunniens* and *Bos mutus* are domestic and wild forms of yak, respectively, and are both native to the Qinghai-Tibetan Plateau and adjacent high-altitude regions (Shi et al. 2016; Chen et al. 2018). Yanglong yak is a new breed bred from Qinghai Plateau Yak and wild yak after long-term natural breeding and artificial mating (Shi et al. 2016). It has been living in cold and hypoxia environment for many years, which has the characteristics of rough feeding tolerance and environmental adaptability. Therefore, this study assembled the complete mitochondrial genome for Yanglong yak from Qinghai Province of China, and investigated its genetic relatedness to other taxa within the subfamily Bovinae (Cetartiodactyla: Bovidae).

The sample selected for this study was a 3-year-old healthy female yak, and the blood sample was collected from Yanglong Township, Qilian County, Haibei Tibetan Autonomous Prefecture, Qinghai Province (38.69°N, 98.55°E), and was used for DNA isolation with the QIAamp DNA Blood Mini Kit (Qiagen, CA, USA). A specimen was deposited at the Key Laboratory of Yak Breeding Engineering of Gansu Province, Lanzhou Institute of Husbandry and Pharmaceutical Sciences (Lanzhou, Gansu Province, China, Xian Guo and guoxian@caas.cn) under the voucher number BMXBG2020053. The genomic DNA was extracted from Yanglong yak, is stored at −80 °C (ultra-deep-freeze refrigerator) in the sample storage room of our department. Library construction and paired-end DNA sequencing with Illumina HiSeq X^™^ Ten Sequencing System (Illumina, CA, USA) were conducted by Annoroad Gene Technology (Beijing, China), which yielded a total of 15.33 M 150-bp raw paired reads. The program MITObim version 1.9 (Hahn et al. 2013) was employed to assemble the mitochondrial genome along with a previously published reference sequence (GenBank accession: JQ692071) (Qiu et al. 2012). The mitogenome annotation was done by comparing with those of its congeners.

The mitochondrial genome of the Yanglong yak (GenBank accession: MT649466) was successfully assembled with an average coverage of 27.1X. It is 16,323 bp long with an A + T-biased nucleotide composition (33.7% A, 25.8% C, 13.2% G, and 27.3% T; ‘light strand’), and encodes the typical set of 37 animal mitochondrial genes (incl. 13 protein-coding, 22 tRNA, and 2 rRNA genes) along with a noncoding control region. Its gene order and codon usage are highly conserved, and are identical to those of the previously published mitochondrial genomes of its congeners (e.g. Hiendleder et al. 2008; Wu et al. 2016; Guo et al. 2019). In all, the 13 protein-coding genes involve two types of initiation codons (ATA & ATG) and three types of termination codons (TAA, TAG & T). The 22 tRNAs range in size from 60 (*tRNA-Ser^AGY^*) to 75 bp (*tRNA-Leu^UUR^*) with a total length of 1509 bp. The secondary structures of the tRNA genes are analyzed and predicted by tRNAscan-SE version 2.0 online website, which is similar to that of other mammals, except that *tRNA-Ser^AGY^* cannot form the typical clover structure due to the lack of the DHU arm. In addition to *tRNA-His*, *tRNA-Ile*, *tRNA-Lys*, and *tRNA-Tyr*, a total of 43 base pair mismatches (34 G-U, 3 A-C, 3 U-U, 2 A-G, and 1 C-U) were found by the prediction analysis of 22 tRNA pairs, about 80% of which are G-U mismatches. The *tRNA-UCN* is the most base pair mismatched gene with 5 G-U mismatches. The two rRNAs are 957 bp (*12S rRNA*) and 1571 bp (*16S rRNA*) long, respectively. The control region is 893 bp long with an A + T-biased nucleotide composition (60.9% A + T; ‘light-strand’). Besides, a 31-bp-long origin of L-strand replication is present between *tRNA-Asn* and *tRNA-Cys*.

To investigate the genetic relatedness of Yanglong yak to the other taxa within the subfamily Bovinae, a maximum-likelihood (ML) phylogenetic analysis was conducted using the concatenated sequences of all 13 protein-coding genes (alignment size: 11,370 bp) with the program PhyML-aLRT version 2.4.5 (Guindon and Gascuel 2003) as implemented in TOPALi version 2.5 (Milne et al. 2009) (Figure 1). The best-fit nucleotide substitution model is ‘GTR + G+I’. Bootstrap support values were estimated from 100 random runs following the method of Anisimova and Gascuel (2006). The outgroup taxa employed in the phylogenetic analysis are three confamilial species from the subfamily Caprinae, including *Hemitragus jayakari* (FJ207523) (Hassanin and Douzery 1999), *Naemorhedus goral* (JX188255) (Yang et al. 2013), and *Ovis ammon* (KX609626) (Mao et al. 2017). The yak in this study was found to be closely related to Datong yak, Pamir yak, Tianjun yak, polled yak, Seron yak, Sunnan yak, a series of Domestic Yak and wild yak, followed by Jinchuan yak and Gannan yak, and slightly far away from *Bison* and *Bos taurus*. The high genetic similarity between domestic and wild yaks was also previously confirmed by Zhang et al. (2009). In addition, it also revealed that the two genera *Bison* and *Bos* failed to form two distinct monophyletic groups, which could be attributed to the frequent hybridization between them (Douglas et al. 2011; Shi et al. 2016).

**Figure 1.**
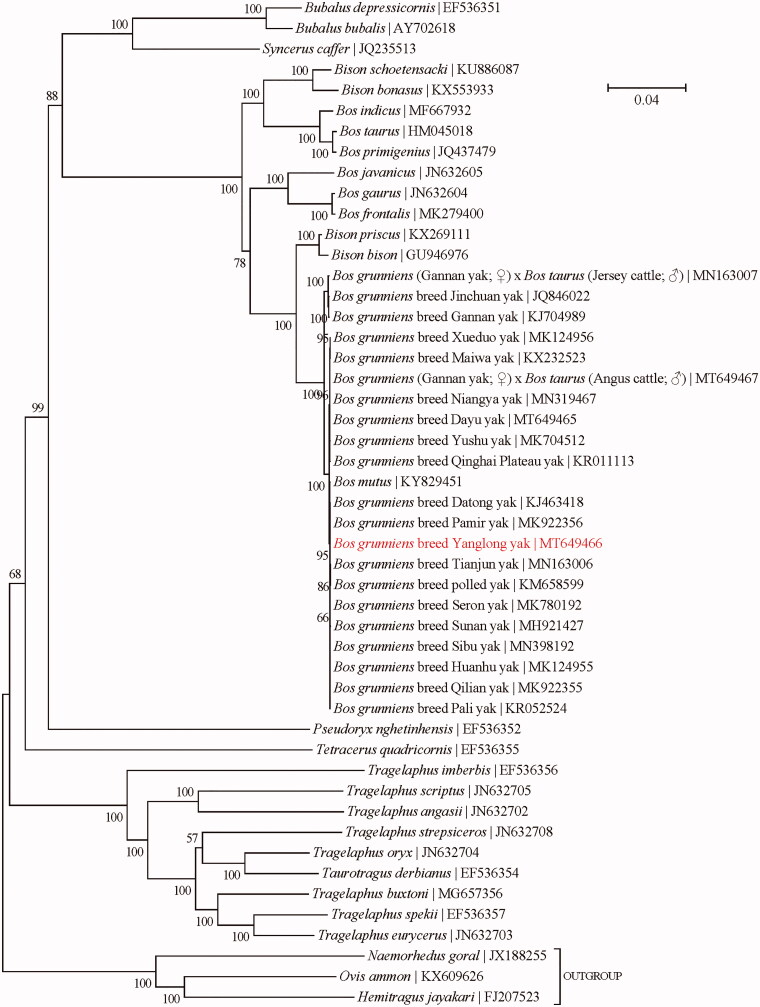
Phylogeny of the subfamily Bovinae based on the maximum-likelihood analysis of the concatenated sequences of 13 mitochondrial protein-coding genes (alignment size: 11,370 bp). The best-fit nucleotide substitution model is ‘GTR + G+I.’ The bootstrap support values next to the nodes are based on 100 random runs.

## Data Availability

The data that support the findings of this study are openly available in GenBank of NCBI at https://www.ncbi.nlm.nih.gov/nuccore/MT649466.
